# Modern Demonology and Divination
*M. Tullii Ciceronis Libri de Divinatione et de Fato.The Course and Tendency of History since the Overthrow of Napoleon I. By Professor G. G. Gervinus.Contributions to Proleptics; or, the Science of Prevision. By Thomas Laycock, M.D. (York). Lancet, 1841-1843.The Coming Struggle among the Nations of the Earth; or, the Political Events of the next Fifteen Years.The Future of the Human Race; or, a Great, Glorious, and Peaceful Revolution near at hand, to be effected through the Agency of Departed Spirits, &c. By Robert Owen.A Review of the Spiritual Manifestations, in which it is attempted to prove that the Spirit-Rappings, &c., are the Work of Evil Spiritual Agents. By the Rev. Charles Beecher (brother of Mrs. Stowe).Table-Moving Tested, and proved to be the Result of Satanic Agency. By the Rev. N. S. Godfrey, S.C.L., of St. Catherine Hall, Cambridge, and Incumbent of Wortley, near Leeds.Table-Turning the Devil's Modern Masterpiece. Being the Result of a Course of Experiments. By the Rev. N. S. Godfrey, &c.Table-Talking. Disclosures of Satanic Wonders and Prophetic Signs. By the Rev. E. Gillson, M.A., Curate of Lyncombe and Widcombe, Bath.Table Turning not Diabolical. A Tract for the Times. By the Rev. F. Close, A.M., Incumbent of Cheltenham.Some Account of the Apparition of the Blessed Virgin at La Salette, in the Diocese of Grenoble, France.


**Published:** 1854-01-01

**Authors:** 


THE JOURNAL

OF

PSYCHOLOGICAL MEDICINE

AND

MENTAL PATHOLOGY.

JANUARY 1, 1854.

### Art. I.—MODERN DEMONOLOGY AND DIVINATION.*

The association of ideas is manifested in national and social as well as individual habits of thought. There is also from age to age a progressive development of nations and societies which, varying in its successive phases, begins and ends in a like manner in every successive cycle, because it is guided onwards by similar associations of ideas, each linked to its predecessor and developing its successor. It follows, therefore, that the stages of development in the lives of nations as well as of

* M. Tullii Ciceronis Libri de Divinatione et de Fato.

The Course and Tendency of History since the Overthrow of Napoleon I. By Professor G. G. Gervinus.

Contributions to Proleptics; or, the Science of Prevision. By Thomas Laycock, M.D. (York). Lancet, 1841-1843.

The Coming Struggle among the Nations of the Earth; or, the Political Events of the next Fifteen Years.

The Future of the Human Race; or, a Great, Glorious, and Peaceful Revolution near at hand, to be effected through the Agency of Departed Spirits, &c. By Robert Owen.

A Review of the Spiritual Manifestations, in which it is attempted to prove that the Spirit-Rappings, &c., are the Work of Evil Spiritual Agents. By the Rev. Charles Beecher (brother of Mrs. Stowe).

Table-Moving Tested, and proved to be the Result of Satanic Agency. By the Rev. N. S. Godfrey, S.C.L., of St. Catherine Hall, Cambridge, and Incumbent of Wortley, near Leeds.

Table-Turning the Devil's Modern Masterpiece. Being the Result of a Course of Experiments. By the Rev. N. S. Godfrey, &c.

Table-Talking. Disclosures of Satanic Wonders and Prophetic Signs. By the Rev. E. Gillson, M.A., Curate of Lyncombe and Widcombe, Bath.

Table-Turning not Diabolical. A Tract for the Times. By the Rev. F. Close, A.M., Incumbent of Cheltenham.

Some Account of the Apparition of the Blessed Virgin at La Salette, in the Diocese of Grenoble, France.

individuals are comparable, and that the history of a deceased body politic may offer lessons of experience to one existent.

We have been led to these considerations by reflection upon the extent to which divination is now prevalent, using that term in the sense of a searching into the unknown present and the future. *Science* has long manifested the glorious characteristic of prescience, and in this, as in every preceding age, we have witnessed examples of foreseeing sagacity the result of mature *experience*. But in addition to these, we have had unusual methods of divination made popular as well with persons of literary attainments and cultivated minds as with the ignorant and superstitious. Clairvoyante youth of both sexes have been used for all those purposes to which divination was formerly applied; and now, more recently, demonology and demoniacal possession have been widely studied and practised. The latter are interesting as a question of psychology, and we propose noticing them the more especially at present; not neglecting, however, those true and natural methods of anticipating the future which are based on philosophy, and therefore exclude the supernatural.

Mesmeric divination will probably be extinguished by this new folly; we need not therefore notice it here.

Mankind has sought in all ages to know its destiny as well in this world as in the next. Every available and conceivable means have been adopted for this purpose,—the most trivial and absurd, the most awful and mysterious. There is no impulse so strong as this instinctive inquiry into the future; none from which more pleasure is derived or by which more anxiety is created. The novel reader is the most familiar illustration of the one, the monomaniac who destroys himself to escape an imaginary future evil is an illustration of the other.

Now what is so general and universal a practice cannot in its nature be wrong. The search into the future may be defended by various arguments, and its propriety and practicability easily established. We might instance our daily experiences, and from the practice on a small scale defend it on the largest. Perhaps, however, the example of the All-wise Artificer of the universe is the most cogent argument in its favour. On every side we see the most manifest and beautiful proofs of that beneficent provision for the future which under the term Divine *Providence*, or foreseeing of and providing for the future, has been recognised as one of the greatest and most lovely attributes of the Deity, and has even become a name for the Deity himself. Looking abroad into the planetary or kosmic creation, we find how nicely balanced in relation to each other are all those mighty masses of matter which sweep through space with such terrific rapidity, and with what a perfection of adaptation to the future do those in more immediate relation to this earth attract and repel it. However certain we may feel as to the future stability of our planetary system, and however confidently we may expect the sun to arise in the morning, yet perfect certainty of the event was only attained in the days of old, after that terrible deluge of waters, by the declaration of God himself. In these later times it has pleased him so to enlighten men's minds that they are more able to comprehend the excellency of his wisdom in the order of his providence. A great and wise king, himself a great naturalist and moral philosopher (and we quote him in deference to the theologians who have entered the field of prescient knowledge), has left on record that the spirit of man is the candle of the Lord. By this "candle" we read that those great globes poised loosely in space, all move in their orbits in the direction most calculated to give them stability; that of all the innumerable arrangements which might have been made, not one could have led to this result more effectually. Laplace, noting this wise provision for the future, has calculated that it is above four millions of millions to one in favour of these motions having been directed by one original or first cause. Certain as we may feel that the sun will rise to-morrow, it is above two million times less probable than the truth of the position that the motions of our system were designed with a distinct reference to their future stability.

Coming to *living* organisms, we again find the example of an admirable foreseeing of and a provision for the future. The student of natural history or of natural theology cannot but be aware of those prospective contrivances for the well-being of living things which encounter him at every step. Long before lungs are required they are ready formed and adapted to future wants; eyes are developed before vision is necessary; ears before hearing; and for many years before it can come into play that most exquisite of all these prospective contrivances, the human brain, is elaborated and made ready for future use.

That which is seen in development, is seen also in the instinctive uses, so soon as the use is necessary, of the machines, instruments, and weapons thus wisely provided beforehand. Philosophers have long recognised the operation of the instinct in lower animals as being something little less than Divine, and perhaps nothing strikes the mind so much as that preparation for the future which marks the instinct for propagation of the species in every kingdom and division of nature, from the highest to the lowest. It is not in the domestic economy of insects only that this admirable regard for the future is manifested, although in these particularly, and the bee and other hymenoptera, it is most curiously shown. The principle pervades the whole series of animated existence, and is the most highly developed in man himself.

The sciences and arts are, in fact, nothing more than the result of the evolution of the instincts into reason and will. Every one and each has its counterpart in the animal economy. Science is nothing more than the knowledge of the necessary order of events; reason is the faculty which enables us to perceive and know this order; philosophy is the right use of this reason in determining such order. Hence, the greatest philosophers have not failed to perceive that man has a special relation to his Creator, inasmuch as he appears to be the only one of terrestrial beings capable of perceiving the wisdom of the Divine Providence, and of investigating the order of creation. It was when impressed with these ideas that Bacon, in his opening aphorism, declared man to be "the minister and interpreter of nature." So also, long anteriorly to Bacon, Epictetus observed—"God hath introduced man [into creation] as a spectator of Himself and His works; and not only as a spectator, but an interpreter of them." It is specially in his interpretation of God's counsel in the past and the future that man is pre-eminent; to *divine* is most emphatically the true characteristic of man's spiritual nature; to "look before and after" is the very essence of all his faculties; and the knowledge of that future existence for which his present is only the preparation, is the culminating point of his knowledge.

It is not, therefore, a matter of surprise that man, in all ages, in all climes, in every stage of political and social development, should follow the corporeal and intellectual instincts of his nature. In domestic life, in agriculture, and indeed in all arts, in politics, in all sciences, he descries and prepares for the future. That is the law of his nature; in following that law, under the guidance of sound principles, man fulfils his duty. The search into the future is therefore in itself laudable and necessary; it is only in the mode in which it may be conducted that there can be error or sin.

Two principal methods of investigation have been adopted by mankind in all ages; namely, the natural and the supernatural. The natural was founded first upon observation and experience; then upon science, or general principles. A simple observer of the phenomena of nature is enabled to foresee the recurrence or occurrence of natural phenomena, although wholly devoid of scientific knowledge. Thus the seaman or shepherd knows well the indications of change in the weather; thus also the course of the seasons and of vital phenomena of an obviously periodic character is manifest to simple observation; neither meteorology, nor astronomy, nor physiology is needed. In morals and in politics, long-continued observation is the foundation of that sagacity which foresees the end of a series of events. It is by this that—

"Old experience doth attain
To somewhat of prophetic strain."

The prediction of events by the aid of science has astrology for its basis; that is to say, astrology, in the true sense of the term, as designating the science of the universe. That at a very early period of history mankind had attained to a very accurate knowledge of the system of the universe is rendered very clear by recent historical researches. The ancient Egyptians and Chaldeans knew the exact length of the solar year, and founded several important periods on that knowledge. In fact, they were not only as perfectly acquainted with the great principles of astronomy as the men of the present time, but appear to have had a much deeper insight into *meteorology*. As to the latter, they were both speculative and practical. Speculative to an extent moderns have not ventured, for they marked out great periods of time, within which cycles of changes would be completed of vast extent and importance. The Annus Magnus, or Great Cycle of the Egyptians, consisted of from 300,000 to 360,000 years. This being completed, the whole assemblage of celestial phenomena which are regarded as the influential causes of all changes in the sublunary world being restored to the same initial order, and proceeding in the same catenation as before, the whole series of events that depend upon them follow in their former connexion of place and time. The same individual men are doomed to be born again and perform the same actions as before; the same arts are to be invented, the same cities built and destroyed. This philosophical theory is probably referred to by the author of Ecclesiastes, or the Preacher, who applied his heart "to know, and to search, and to seek out wisdom and the reason of things," when he remarks, "The thing that hath been, it is that which shall be; and that which is done is that which shall be done; and there is no new thing under the sun. Is there anything whereof it may be said, See, this is new? It hath been already of old time which was before us." According to Mr. Cullimore, the Chaldeans represented the origin and destiny of the world as depending upon two grand astral conjunctions, on the occurrence of which, in a peculiar season of the year and point of the heavens, it was fated to perish by a deluge or conflagration. These doctrines were very widely diffused through the whole of the East, and appear to have passed from thence to Greece on the one side and South America on the other, having been traced in the mythology of the Aztecs. But, although our first convictions would lead us to consider these views as entirely speculative, yet as to the astronomical portion of them we are not without some indications of foundation in truth. First, as to the Annus Magnus of 360,000 years, we may remark that, knowing how much the number 7 was used by the ancient astronomers and philosophers, we resolved to try, by way of experiment, what astronomical cycles would produce, if multiplied by 7, an approximation to the number of years constituting the Annus Magnus. Singular to say, the first cycle tried produced the number very nearly. The student of astronomy is well aware of what is termed the precession of the equinoxes; namely, a motion of the equinox on the ecliptic, by which it constantly travels backward or retrogrades, but at such a very slow pace that it requires 25,868 years to complete the tour of the ecliptic. Multiply this number by 7, and the result is 181,076, being the half (or a little more) of the great cycle of 360,000 years. We next looked for an astronomical cycle of which this might be the half, and which might (like the precession of the equinoxes) correspond to the Egyptian and Chaldean speculation. There is one of this kind of a very interesting character. The earth's orbit at present is *elliptical*, but in some far distant age it was *circular*, and in some far distant age it will be circular again; for it is so kept in a continual state of change by the action of the planets on the earth, that its eccentricity is, and has been from the earliest ages, diminishing, and this diminution will continue (there is little reason to doubt) until the eccentricity is annihilated and the earth's orbit becomes a perfect circle; after this it will again open out into an ellipse to a certain moderate amount, the eccentricity will again increase, attain a certain moderate amount, and then again decrease as before. The time required for these evolutions (Sir F. Herschel states), though calculable, has not been calculated farther than to satisfy us that it is not to be reckoned by hundreds or by thousands of years. Now it is by no means improbable that this cycle may be the Annus Magnus, as it presents its essential characteristics; namely, recurrence of identical planetary relations. It is a remarkable coincidence that the knowledge of this cyclical change was acquired indirectly through ancient Chaldean records of lunar eclipses. By comparing the times of their occurrence with those of the modern era, Halley discovered that which is known as the secular acceleration of the moon's mean motion. The explanation of this acceleration was a puzzle until Laplace discovered further that it was caused by this cyclical eccentricity of the earth's orbit. It is curious, too, that the *embolismal* period of the Egyptians, of 1440 years—a period during which the twelve months became intercalated in the calendar in succession—is a multiple of the cycle. The Chaldeans used another cycle of interest; namely, the soli-lunar period of 600 years, the square of which makes up the *Annus Magnus*. Now the solar year, which is the unit of this Chaldean period, is exactly 365d. 5h. 51m. 35s. 2th. long; it also contains exactly 7421 lunations, of 29d. 12h. 44m. 2s. 48th.—striking proofs of the refined mathematical as well as astronomical science of that wonderful people. How far they were acquainted with geology we know not; but it cannot but strike the mind that this science corroborates the Chaldean speculations, inasmuch as it distinctly points to the occurrence of vast changes in the earth's crust as having occurred at long intervals of time, and caused at one time by mighty volcanic agencies, at another by overwhelming irruptions of the ocean on the dry land, or deluges and conflagrations.

We can hardly conclude that these cycles, so far as the history of mankind is concerned, were otherwise than speculative. It is certain, however, that astronomy and meteorology were practically applied to the daily concerns of life by these Chaldean philosophers. This is manifest both from various passages in the Scriptures and in ancient writers, and from the records of Egyptian and Chaldean science, discovered in the ruins of ancient cities in Egypt and Mesopotamia. Many of the inscribed bricks and inscriptions on stone are almanacks, or seem to contain the data for determining the annual inundations. A passage in Isaiah points to a distinct class of scientific men amongst the Chaldeans, whose duty it was to prognosticate. The entire of the 47th chapter indicates the high state of luxury and knowledge attained by the Chaldeans; but the 13th verse runs—

"Let now the astrologers, (or, marginal reading, 'viewers of the heavens,') the star-gazers, the monthly prognosticators, (or, in the margin, 'that give knowledge concerning the months,') stand up, and save thee from these things that shall come upon thee."

Cicero, in his 'Treatise de Divinatione,' mentions "the auguries not of divine impulse but of human reason, for they predict future nature,—as floods of waters, and at some time a future conflagration of the heaven and the earth." He also classes with these the advice to the Lacedæmonians of Anaximander, the natural philosopher, who warned them to leave the city and their houses, and go armed into the plain, because an earthquake was imminent; and that forthwith it took place, the whole city was overthrown, and a projecting portion of the hill Taygetus thrown down. Cicero also quotes Pherecydes, the teacher of Pythagoras, as pronouncing an earthquake approaching, from the condition of the water drawn from a well. This natural divination, Cicero further shows, can be attained by a knowledge of the chain of causes, —of why those which are past occurred, why those which are now became, and why those which follow will come. If "mortal man," he observes, "could comprehend the entire colligation of all causes, he could be deceived in nothing, for if he knew the causes of the future, he would, of necessity, know the future itself."

There is a regular sequence of events in the life of every living organism, as well as in the starry heavens. The order of this sequence was also specially studied by the Egyptians and Chaldeans. They divided the series of periods into *steps*—hence the *climacteric* periods of life, known and recognised by the Chaldeans, and according to

Aulus Gellius, so named by them; what is more interesting is, that the Druids were as learned as the Chaldeans in these matters. Julius Cæsar, in 'Commentaries de Bello Gallico' [lib. vi. cap. xiv.], says: "These profess to know the size and form of the earth and the world, the motions of the heaven and of the stars, and what the gods will." According to Diodorus Siculus, they studied physiology (*sic*) and ethical philosophy.

But the ancients occupied themselves in that other important branch of prognostics which foresees the origin, progress, and destiny of nations. Cicero mentions this specially as one of the natural auguries, and illustrates it by the example of Solon, who predicted, long before, the rise of despotic government in Athens, just as Napoleon is said to have declared that in thirty years Europe would be either Cossack or Republican—a prediction which might have been verified if the balance of power had not rested with the powerful constitutional monarchy of this kingdom. Now, this natural and philosophical, and therefore true, method has been so corrupted and lost, that it can hardly be recognised amidst the quackeries which have sprung out of it. Astrology, as now practised, is the chief in modern times; the practice of augury was the most frequent in ancient times. These quackeries, however, were so closely mixed up with those of the supernatural class, that they rather belong to it than the former.

The first and only true system of knowledge of the future of the *supernatural* kind is contained in the prophecies of Holy Writ. All others are spurious. It is hardly possible to conceive to what an extent divination, sorcery, magic, and demonology constituted a part of the social system of the ancient nations. Divination was, in truth, a part of their *established religion.* Cicero informs us that the Persian Magi congregated in the temple for the purposes of discussion and mutual instruction, and that no one could be king of Persia who had not been trained in the doctrine and teaching of the Magi. In speaking of the fixed customs, in this respect, of the Athenians, Lacedæmonians, &c., he remarks,—*Omitto nostros qui nihil in bello sine extis agunt, nihil sine auspiciis domi habent.*

We have seen that science and philosophy had their share in the system of prognostics; it must be added, that superstition had more. The ancients relied much on *spiritual* agencies, or on what seemed to them to be such. The greater number of their practices arose out of those curious operations of the nervous system, which in modern times are again betraying man into necromancy, witchcraft, and demonology. The Greek word for a soothsayer or divining priest —*mantis*—is derived from the same root as *mania.* To this day, indeed, in Asia Minor, the idiotic and insane are thought to be inspired.

Such, also, was the case in ancient times; and delirious or clairvoyant girls, known as a Pythian priestess or a sybil, ruled the polity of great, warlike, and learned nations, and accumulated untold wealth. The oracle at Delphis received rich gifts from every quarter of the then civilized world, and had a reputation for truth beyond suspicion. Portents of all kinds, false and true, had their weight; the images of the gods were seen to weep, to perspire, to bleed; were heard to utter warning sounds. Meteors with thoughts of fear affrighted nations, and the occurrence of a fit of epilepsy dissolved important political assemblies. Divination by dreams, or oneiromancy—by departed spirits, or necromancy—by the divining rod, or rhabdomancy—by second sight—by various minor methods, as crystals, a cup—"is not this it in which my lord drinketh, and whereby, indeed, he divineth?" said the Egyptian steward of Joseph—was of ordinary occurrence. Never was the world so given up to a multifarious system of fraud and falsehood as at the Christian era.

When the practice of divination was, apparently, the most firmly established, and when those ideas from which it originated had attained their culminating point in the deification of the Roman emperors, the whole social fabric of the age was nodding to its fall. Ancient civilization had also reached its climax, and the barbarous tribes of the then known world were beginning to press on its confines. Christian civilization seems now to have attained an analogous stage of development. The most ensanguined conqueror of Christendom has the day of his beatification commemorated by a large section of the Christian church; while the despotic ruler of another large section, made up of an agglomeration of barbarous tribes, draws the sword of conquest in the name of religion, and not only claims to be, but is *acknowledged* by millions of Christians to be actually God's vicegerent on earth. In the more civilized nations of the West, dignitaries of one section of the Christian church, and priests of another, stamp with the seal of their authority legendary stories and lying fables, such as emphatically characterized the pagan era; and—worse perhaps than all—men whose education and attainments entitle them to rank with philosophers, corroborate with their authority the superstitious follies of the age.

It may be alleged that our description is overdrawn. It is not so; and to show its accuracy, we will compare briefly the modern with the ancient divination.

The village of La Salette is situated among the mountains, at about four miles from the little town of Corps, which lies below on the high road between Grenoble and Gap. About four miles from the church of La Salette, higher up in the mountains, in the hollow of a ravine which cuts through a table-land bare of trees and rocks, on September 19th,

1846, two little children were watching their cows, the one a boy aged eleven years, the other a girl aged fourteen years. The Blessed Virgin, we are told, selected this time and place to communicate with these children, as " the bearers of a warning message to ' her people,' and to be the depositaries of some mysterious secrets." Shortly after the hour of noon, they saw a " brightness," and a lady in it, who was sitting down with her head in her hands. She spoke to the children. With tears running down her face, she informed them as to various matters touching the present and the future. She reproved the Sabbath labour of the country-folk, and the swearing of the men as they drove their carts; only a few old women went to mass, the rest working during the Sunday all summer; and " during Lent they go like dogs to the butchers' stalls." For these sins she declared there should be no potatoes at Christmas. But we will record the very words of this prophecy.

" The disease shall last; so that this year, at Christmas, there shall be no potatoes at all.

" If you have any corn you need not sow it; all that you shall sow shall be eaten by the animals; or if any does grow up, it shall fall in dust when you thresh it.

" There shall come a great famine.

" Before the coming of the great famine, the children below seven years of age shall have convulsions, and shall die in the arms of those who hold them; the rest shall do penance by hunger.

" The nuts shall become bad; the grapes shall rot.

" If men will be converted, the rocks and stones shall be changed into heaps of corn; and potatoes shall be sown all over the land."

The " lady" then inquired about their prayers, talked a little small-talk, and at last glided away along the tops of the blades of grass.

" Then she looked up to heaven, then down to the earth; then we could not see her head any more; then we could not see her arms; and then we could not see her feet any more. We saw nothing but a brightness in the air, and soon the brightness went away also."

In answer to inquiries how this " lady" was dressed the children replied:

" She had on white shoes, with roses about her shoes. The roses were of all colours. Her socks were yellow; her apron yellow; and her gown white, with pearls all over it. She had a white neckerchief, with roses round it; a high cap, a little bent in front; a crown round her cap, with roses. She had a very small chain, to which was attached a crucifix; on the right were some pincers, on the left a hammer; at the extremities of the cross was another large chain, which fell, like the roses, round her handkerchief. Her face was white and long."

In addition to what we have mentioned, the lady confided to each of these children a secret which he was not to tell to the other. As to these, they have hitherto maintained an impenetrable silence, excepting in confiding them to the Pope, to whom they were transmitted in 1851.

"During the past year 1851, the children, in the presence of certain persons named by the Bishop of Grenoble, wrote each on a sheet of paper, which was folded and sealed by the writer, the secrets entrusted to them. The Bishop then directed M. Rousselot and another priest to carry these sealed packets to Rome, and deliver them into the hand of the holy father. This was done. His holiness first broke the seal of the one, and read it without making any remark. On perusing the other, he walked with it to the window, and after having read it said: 'It is not only France that has sinned, but Germany, Italy—all Europe!' When M. Rousselot went to take leave of Cardinal Lambruschini, the cardinal said—'I know the secret; the holy father has confided it to me.'"

If these two "augurs" laughed at each other, as their Roman predecessors would have done, the fact is not chronicled.

The water of the fountain became, of course, a means of cure for diseases, and worked many wonderful miracles, both at the spring and when taken to a distance. Innumerable pilgrims visited the scene of the vision. Their feet wore away the herbage, their hands tore up the earth and morsels of stone; fourteen crosses were erected along the line which "the lady" traversed; they were hacked and cut daily by the "faithful," 60,000 of whom ascended the mountain in one day.

Official cognizance was taken of the whole affair. Ecclesiastics of rank visited the "holy mountain;" amongst the rest the present Bishop of Orleans, M. Dupanloup, who could not refrain from saying to himself continually, "It cannot be but that the finger of God is here!" but who, however, frankly acknowledges that both the children were very disagreeable, vulgar, and habitually rude (the boy, we are also told, was an habitual liar), reciting their story like a lesson, and very guarded in what they said about it. The credulous (?) bishop gives us the outlines of the cross-examinations to which he subjected them, and examples of questions put to them. We find that the questioners did not hesitate to tell them lies, so as to entrap them; as, for example, that the Virgin had told the secrets which she had confided to them to a "holy nun;" that the Pope was "greater" than the Blessed Virgin, &c. When it was hinted to them that their visitor might have been the devil, they promptly replied, "But the devil does not wear a cross!"

The visionary tales of these children have been widely spread, and as widely credited by "Catholics." Churches are being built in various countries in honour of Our Lady of Salette, and even a confraternity is established in England bearing her name. The cures she has wrought are as numerous and as well authenticated as those of the most popular quack medicine, the testifiers being *bishops* of the Roman church.

The parallel to this history is not wanting in pagan religious history; such visions were by no means uncommon, so that the difficulty is rather in the selection. That which gave origin to the established religion and priests of ancient Italy, namely, augurs and divination by auguries — a religion which governed the entire polity of ancient Rome—is, perhaps, the most interesting to us. Cicero gives us the particulars:—

"It is said that a certain Tages started suddenly from the earth, as the ground of a Tarquinian field was being ploughed, and a deeper furrow than usual made, and addressed the bullock-driver. This Tages, as is stated in the books of the Etruscans, appeared in form to be a boy, but in wisdom was an old man. The bullock-driver, being astonished at his appearance, raised a loud cry of astonishment, and a concourse of the people being thus excited, in a short time the whole population of Etruria assembled on the spot; that he then said many things to numerous hearers, who listened to his words and committed them to writing; that, further, this address comprised the science of divination, and that it has been added to by new knowledge based on the same principles. This science we have received from these persons; these writings are preserved; this is the source of the science. Now is there anyone, I will ask, so silly as to think that there was ploughed out either a god or a man? If a god, why should he, contrary to his nature, hide in the earth, so that when exposed by the plough he might behold the light? Why, indeed, could not this same god communicate science to men from a loftier spot? But if this Tages was a man, how could he live covered over with earth? or where could he have learnt that which he taught to others?"

"But," Cicero significantly adds, "I should indeed be more foolish than they who credit these things if I seriously argue the matter." Equally absurd would it be to attempt to demonstrate, in reference to "Our Lady of Salette" what is obvious enough to any but the most obtuse understandings.

The portents of the bleeding picture of Rimini, and the perspiring and the weeping statues of the saints elsewhere in Italy, have equally their pagan parallels; nothing seems changed. It is recorded that at the commencement of the Massican war the images of the gods perspired, blood flowed in a river and rained from heaven, mysterious voices were heard, &c., &c. These follies Cicero ably combats in terms perfectly applicable to similar modern portents. That the augurs deliberately practised deception is certain, both from various facts and from antiquarian researches. The sacrifice of an ox in which the augurs could find no heart, is an anatomical portent closely parallel to that well-known Roman-catholic portent, the liquefaction of the blood of St. Januarius.

The populace of the ancient Italian and Grecian cities were excusably credulous, for they were almost wholly uneducated. A great proportion were enslaved; and it was then (as now in the slave-holding states of North America) a capital crime to teach a slave. Natural history and physiology were so little advanced, that even the educated had no aid from science in the detection of the frauds of the priesthood, or in the explanation of those natural phenomena to which the expounders of the established religion gave a portentous and religious character. The established faith on these points was also as intolerant then as now: for it is on record that Anaximander was condemned to death for impiety, which consisted in referring to natural causes the so-called auguries. In modern days the populace are as uninstructed as in the ancient in that knowledge which would dispel their superstitious terror, and prevent them being the dupes of religious frauds; they are therefore excusable also. But what shall we say of the priesthood and educated laity of the Roman-catholic church from the Pope and Prince downwards, who not only wink at, but expressly sanction, multitudinous fables like that of Our Lady of Salette?

The representative faculty of man has ever and in all times strangely clothed in visible forms that fundamental idea of the spiritual and invisible which is inherent in his nature. In the imaginative south the material representations have little changed as to the form and substance; only the name and a few non-essential characteristics have been modified. In the more metaphysical north, ideas of spiritual agency of a gloomy character have been predominant, as with the ancient Hebrews, the Etruscans, the Chaldeans, and the races of the east. The representation of the instincts and emotions has in the sunny south developed itself in the various forms of love, especially the physical and maternal; in the north, the sterner instincts and feelings have predominated. Be this as it may, demonology, necromancy, and witchcraft, although not by any means exclusively the pursuits of the races just mentioned, are less modified by the beautiful and the pleasurable than in Greece and Italy. The popular taste for table-turning, spirit-rapping, and demonological researches, is only another manifestation of that series of ideas which two or three centuries ago took the form of witch-finding; which anteriorly to that age occupied the clergy in exorcisms with bell, book, and candle; and which in the ancient nations of the east led to the theory of demoniacal possession, and raised up the profession of exorcists. Egyptian, Assyrian, Etruscan, Grecian, and Roman literature and remains sufficiently show how widely the doctrines of demons, and of a spiritual being powerful only for evil, influenced the mythology, religious ceremonies, and acts of those people. The term demon amongst the Greeks, at least, did not necessarily imply an *evil* spiritual being, as it appears to have implied amongst the Jews.

With both, however, persons affected with epilepsy or epileptic-like diseases, were thought to be possessed by 'demons.' Hence the term *morbus sacer*, as applied to it; and hence the term comitial disease amongst the Romans, with whom it was a law that if a person were attacked with epilepsy during a meeting of the freemen, it was forthwith dissolved. In the Hippocratic writings, a little essay on the Sacred Disease gives us an interesting glimpse into the philosophy and popular superstition of the day. The writer, describing the practices of the professional exorcists of the age, remarks—

"If they [the epileptic] imitate a goat, or grind their teeth, or if their right side be convulsed, they [the exorcists] say that the mother of the gods is the cause. But if they speak in a sharper and more intense tone, they liken this state to the horse, and say that Posidon [Neptune] is the cause. Or if any excrement be passed, which is often the case owing to the violence of the disease, the appellation of Enodius [Hecate?] is given; or, if it be passed in smaller and denser masses like birds', it is said to be from Apollo Nomius. But if foam be emitted by the mouth, and the patient kick with his feet, Ares [Mars] gets the blame. But terrors which happen during the night, and fever, and delirium, and jumpings out of bed, and frightful apparitions, and fleeing away—all these they hold to be the plots of Hecate [the queen of Hades] and the invasions of the heroes [daimons], and use purifications and incantations," &c. &c.

The casting out or exorcism of these daimons was amongst the Jews also an ordinary practice, as is evident from texts in the Holy Scriptures, both old and new. Allied to this notion of spirit possession, was that of having a "familiar" spirit, or a spirit of prophecy, at command, a "python," &c. The persons possessed by a spirit of prophecy were generally females; they suffered from diseases of the nervous system, especially somnambulism, catalepsy or trance, and hysterical delirium. They were used by their masters for the purposes of gain, as has been done only very recently by the masters of clairvoyante girls in this country, on the Continent, and in the United States. As respects these, indeed, the analogy is complete; as respects the devil and departed spirits, it is a fortunate thing for the admirers of spirit-rapping and table-turning that the analogy is not complete, and inanimate wood is possessed by them rather than living men and women.

Our demonological sketches might be much extended, but this short introduction will help us to understand the modern development of this folly, as it is set forth by a certain reverend divine of the Church of England, with whom we must in justice associate another erring brother, the Reverend E. Gillson, of Bath. This gentleman has largely helped in developing "the very strange excitement, and the very sinful practices" which the publication of the Satanic theory of tableturning has caused in Bath. As Mr. Godfrey is, however, primarily and more directly identified with the "Satanic theory," we limit our notice to his little publication. Mr. Godfrey takes his stand upon "God's Word," that is to say, upon the facts recorded in Holy Writ. By collating these facts and the various doctrines of Satanic agency scattered through the inspired volume, Mr. Godfrey satisfactorily (to himself) proves that the ancient Egyptian magi (or natural philosophers) were enchanters in the true sense of the term, that is to say, that the magic whereby they imitated the miracles of Moses was not a natural magic, such as is practised in modern times by the professed conjurors, whether European, African, or Asiatic, but a supernatural. This supernatural agency was spiritual; being not of God it was opposed to God, "and as the only supernatural agency *opposed* to God is the Satanic, *we* say, therefore, *they* [the enchantments] were performed by Satanic agency." This is an example—the first we meet with—of Mr. Godfrey's mode of argument. He quotes Balaam (a magos) as another enchanter; also the witch of Endor (a necromancer), who, according to Mr. Godfrey's own showing, was identical in her character with the damsel having the spirit of divination, mentioned in the Acts (xvi. 16), "which brought her master much gain by soothsaying," and this identical damsel, who had a spirit of Python, leads to the Pythoness proper of Greece, who was a ventriloquist like the Endor dame. Having identified the three as being similar, Mr. Godfrey comes to the conclusion—not that the Greek Pythoness was an impostor—but really inspired by Satan!

This perversion of intellect is so characteristic of this whole class, and is itself so interesting a fact in the psychology of these follies, that we quote Mr. Godfrey's own views:—

"We have thus, therefore, a clear connexion established between the Python of the heathen oracles, the witch of Endor, and the 'damsel' in the Acts. From the witch of Endor we learn that the Hebrew OHV [*sic*] is rendered by εγγαστρίμυθος, or 'speaking out of the belly.' From the history of the heathen oracles, we learn that the Python or Pythoness was called by that name used in the Septuagint, because she spoke from the belly; and from Acts we find that the spirit of Python was an evil spirit, which Paul commanded, in the name of Christ, to come out, and which at his command 'came out at the same hour.' In all these, therefore, we can clearly trace the existence of a supernatural agency; that agency is, by inspired writ, evil spirits. Satan is the prince of the evil spirits, and therefore this agency is Satanic. I am not about to speak of mesmerism, else I conceive I have all the ground-work prepared to show that mesmerism is similar to this, and that by whatever agency the one was effected, the other is, inasmuch as the results are almost identical; and that if evil spirits were the hidden actors in ancient divination, so are they in modern mesmerism."

There are those who have attempted to explain the phenomena which Mr. Godfrey holds to be of Satanic origin, by investigation of the modes of action of the nervous system, and of the phenomena themselves, considered as physical phenomena. That attempt has succeeded. Mr. Godfrey, however, is no child in the use of the well-worn weapons of theological controversy. He first identifies his absurdities with the great truths of Christianity; then he places the origin of these truths, and therewith his absurdities, "beyond the limit even of philosophic imagination." They "can be realized by faith alone; and in such an investigation, the little child, taught by God the Holy Ghost, is far wiser than the grey-haired philosopher, in whose brain is accumulated the wisdom of ages." He hesitates not for a moment to assert, by implication, that he is such a simple man so inspired; or that, in relation to modern philosophers, he is like Paul preaching to the philosophical Athenians. Daily experience, in his view, establishes "the fact that many thinking minds—minds of undoubted power and talent—are, for all practical purposes, *infidel*"—the infidelity apparently being a disbelief in Mr. Godfrey's demonology. Reason, of course, is out of the question; reason is therefore "the word with which the devil baits his snares, which he sets to catch souls." Science must stop on the borders of the natural, which borders Mr. Godfrey undertakes to fix, and hand over to faith (*id est,* to his wandering imagination) the investigation of the supernatural. And so Mr. Godfrey—helpless man, with his philosophical faith, proceeds to exercise *his* reason with *experimental* researches into the philosophy of the supernatural.

We do not propose to follow Mr. Godfrey through these researches: it would be only to waste our readers' time, and occupy valuable space needlessly. Mr. Godfrey, prostrate on the floor, beside the alphabet-board of his national school, ready to spell at the devil's dictation, is only a subject for the satirist, and to which a Molière is the man to do justice. His curate seems to have aided him; also his wife, maid-servant, divers "ladies," &c. He examines and cross-examines the "evil spirit," who answers his questions affirmatively, by lifting a table-leg; negatively, by remaining still. These are examples of the sort of questions put, and the answers given:—

"Are you an evil spirit?—Yes.
"Are madmen possessed by devils?—Yes.
"Is epilepsy possessive?—Yes.
"Are you the spirit of a dead person?—Yes.
"Have you power to come into and to leave this table?—Yes.
"Does the devil send you here?—Yes (emphatically).
"Does he send you for the purpose of deceiving us?—Yes (very decidedly).
"Should you like to come again?—Yes (emphatically).
"Will you come again, if I summon you in God's name?—Yes."

These are some of the questions put, and the contradictory answers given, by this supposed spirit of a table. The "spirits" were, of course, successfully exorcised in the name of Jesus by this weak-minded man, who, imitating Christ, commanded them, in his name, to "quit the table"! What is very singular is, that the spirit being by its own confession an inveterate liar, what was stated was unhesitatingly believed, when it spoke of the spirit world, of Satan, of the palsied, insane, and epileptic as being possessed.

It is proverbial that the devil can quote scripture, but Mr. Godfr y undertakes to make him *prove* what he thinks scripture. It is ve y well known, and beyond doubt, that *some* of the cases, at least, of demoniacal possession described in the New Testament were simply cases of epilepsy and insanity. This Mr. Godfrey evidently feels to be a weak point in his demonological system, and he justifies it thus:—

"On being asked whether the unclean spirits ever entered into any one, he knocked an affirmative; and when asked, 'Into whom? What diseases were possessive?' he spelt 'MADMEN,' 'FALLING SICKNESS' (or epilepsy), 'Palsy,' 'Murder.' We also learned from him that there were good angels, that he could see them," &c.

Mr. Godfrey gives his "explanation" of the phenomena, that is to say, the deductions which he has drawn from his experimental researches. This effort of his "reason" is as follows:—

"The placing the hands on the table is a sort of incantation. By it the sitters signify their wish to be brought into communication with the spirit-world. They sit until they are observed by some one of the 'wandering spirits,' who thereupon enters the table, making it crack at the moment of its entering in. The reason why it will not obey any commands, *unless hands are placed on*, has suggested an idea which, if it be true, is a very solemn one. It occurred to me, while writing, that the table 'moved simply by the laying on of hands.' L. H. (before referred to) says: 'This moral nothing . . . . *on the imposition of your hands* BEGINS TO LIVE!' Can it be that this is the beginning of Satan's last struggle, that *on the imposition of hands*, the table is endued with power *from the devil*, as the Lord's servants, *on the imposition of hands*, were, *in the Apostles' days*, endued with power *from on high?* I merely ask, Can it be?"

The hint, that "this is the beginning of Satan's last struggle," points to the idea which in fact overlays all others in the mind of Mr. Godfrey and his disciples. It is, that the predicted days are at hand when Satan will make his "last grand effort to overthrow Christ's kingdom," days "to which the eye of every student of prophecy is directed." He adds:

"I am now firmly convinced that table-moving is a Satanic device; these various manifestations indicate that the enemy is growing bolder; the bolder he becomes, the more open will be his miracles; the more open his miracles, the closer our proximity to the development of the antichrist, ruling by Satanic possession and power. And, oh! if the tribulation of those days shall be of so awful a character, as it shall be, 'that except those days should be shortened, no flesh should be saved,' " &c.

We have seen that tens of thousands gave full credence to the legend of Our Lady of Salette; so it is with this delusion of spirit-rapping and table-talking. The excitement in the United States has been so great that numerous cases of insanity have resulted. One writer on this subject (Thomas Henry Spicer) states that there are not fewer than thirty thousand recognised *media*, or persons having, in biblical phrase, familiar spirits, in various parts of the United States. In the city of Philadelphia alone it is asserted that there are no fewer than three hundred magnetic circles, holding regular meetings, and receiving communications—circles composed of members of highly respectable families. The Rev. W. C. Magee has published a sermon which he preached at the Octagon Chapel, Bath, with the view of arresting the increasing excitement on the subject in that city.* The Rev. F. Close also beats the "drum ecclesiastic" at Cheltenham in his "Table-turning not Diabolical," a tract less sensibly written than Mr. Magee's. Mr. Close remarks that—

"Those who have watched the bias and tendency of the minds of spiritual people during the last twenty years, will be prepared to expect that anything of the wonderful or mysterious will be readily received as supernatural and miraculous. The character of a large portion of our popular religious literature during the period referred to, whether issuing from the High Church or Low Church press, has had a tendency to create a morbid expectation of wonders; undefined and ardent speculation has been created; a disposition to catch at every trifling event as the fulfilment of some prophecy, according to the private interpretation of some popular SEER!" &c.

All this is true; but it is not true that "philosophical and scientific persons," and they who are "deeply read in the well ascertained facts of experimental and practical philosophy," are "specially prone to scepticism," as Mr. Close asserts. It would be well, indeed, if the spiritual guides of the people were themselves better taught in the laws of inductive philosophy, and especially in experimental and practical research into the physiology of the nervous system. Public decency would then be less frequently outraged, and less scandal and shame brought on the doctrines and example of our Blessed Saviour. As matters are going on, there is indeed great reason to fear that a wide torrent of infidelity and scepticism will sweep over Christendom.

We have one more illustration to adduce of this strange tendency, in

* Talking to Tables a Great Folly or a Great Sin; being the substance of a Sermon. By the Rev. W. C. Magee. Fifth Edition.

modern times, to divination, and then we have done. From a very early period dreams have had a prophetic character attributed to them, and ONEIROMANCY has ever been an important branch of divination. Perhaps Holy Writ presents us with the greater number of illustrations of this practice amongst the Egyptians, Chaldeans, and Jews. It is obvious that the interpretation of dreams entered into the natural philosophy of those nations, and was a branch of learned inquiry. Physiologically it was allied to somnambulism and clairvoyance, the latter being only dreaming under another form. Technically, however, it constituted the "seeing of visions." It is now well known to those acquainted with the physiology of the nervous system, that dreams and visions are but the results of cerebral action, modified by the action of sleep; that they are thoughts presented to a consciousness from which the external world is shut out; and that, for this very reason, they are often *accurate* thoughts, and constitute correct *deductions* as to the past, the present, and the future. It is the nature of dreaming, however, that thoughts thus presented to the mind do not appear as thoughts, but as occurrences and realities; so that an anticipation of a future event, presented to the mind as a dream, appears to the dreamer as if it were occurring. In this way dreams may be, and undoubtedly are, occasionally prophetic, so that oneiromancy is not a method of divination altogether devoid of foundation, and thus the magi may occasionally have given a successful interpretation to a dream.

The prophet Daniel was a magos or philosopher of high rank in the Chaldean hierarchy. He had, moreover, the knowledge of the true God, and was a religious man in practice as well as theory. He "had understanding in all visions and dreams." On the occasion of his discovery of Nebuchadnezzar's dream, he was made "chief of the governors over all the wise men of Babylon," or "master of the magicians, astrologers, Chaldeans, and soothsayers;" and was doubtless highly accomplished in literature and philosophy, being "skilful in all wisdom, and cunning in all knowledge, and understanding science." That he was also an eminent politician may be inferred from the fact, that of the presidents appointed by Darius over the hundred and twenty princes of the kingdom, he was the chief or the prime minister. The visions of Daniel, Ezekiel, and St. John are wonderful prophetic records, in whatever way we view them. It is just now a favourite pursuit of enthusiastic clergymen and pious laymen to guess at the events predicted, and especially to apply the predictions to the present times. Amongst these is a greater oneiromancer than Daniel, and that one is the author of the "Coming Struggle," for he professes to interpret with absolute accuracy the visions of these prophetic seers.

The author of the "Coming Struggle" is a political prophet; and the gist of his prophecies is that—1. The Emperor of Russia will seize Constantinople, and overthrow Turkey. 2. That France will overthrow Austria, and annex its territory, and that with Austria the Papal power will fall. 3. That when the continent of Europe is thus weakened by war, the Emperor of Russia will extend his empire over it, and the huge image, which was the great idea in Nebuchadnezzar's dream, will be complete in the subjection of the territories comprised in the Babylonian, Persian, Grecian, and Roman empires to one man. Britain will, however, escape, and rapidly extend her Eastern empire, and include in it Egypt, Syria, and Palestine, the latter being colonized by Jews. War then breaks out between Great Britain and the Russian Emperor; the United States come to the assistance of the Anglo-Saxons. The countless myriads of Europe under the Russian banner are assembled beneath the walls of Jerusalem in battle array; opposed to them are the battalions of the United Anglo-Saxons intermingled with the Jews, and all is ready. The finale is the Battle of Armageddon, which we give in the writer's words:—

"That awful pause which takes place before the shock of battle, reigns around; but, ere it is broken by the clash of meeting arms, and while yet the contending parties are at a little distance from each other, a strange sound is heard overhead. The time for the visible manifestation of God's vengeance has arrived, his fury has come up in his face, and he calls for a sword against Gog; throughout all the mountains 'tis the voice of the Lord that breaks the solemn stillness, and startles the assembled hosts. The scene that follows baffles description. Amid earthquakes and showers of fire the bewildered and maddened armies of the Autocrat rush, sword in hand, against each other, while the Israelites and their Anglo-Saxon friends gaze on the spectacle with amazement and consternation. It does not appear that they will even lift their hand against that foe which they had come so far to meet," &c.

The fact being that the "foe" meets the fate of the Kilkenny cats.

We remember something like this in Homer's Iliad:—

"Then Jove from Ida's top his horror spreads;
The clouds burst dreadful o'er the Grecian heads;
Thick lightnings flash; the muttering thunder rolls;
Their strength he withers, and unmans their souls;
Before his wrath the trembling hosts retire,
The god in terrors, and the skies on fire."

After the battle comes the millennium, as a matter of course.

It is instructive to note with what a calm conviction of superior knowledge the author of this exposition sets aside the interpretations of "the authorized interpreters of God's revelations." "Dr. Thomas, of America, was the first to find the key," however; the expositor takes that key and unlocks the sacred record.

This brief outline of the psychological position of the public mind must suffice for the present. Our space will not allow us to go into a detailed comparison of the present stage of national mental development with the past stages of nations extinct, however instructive it might be. The well-read student in history will not have much difficulty in drawing the parallel for himself; and the drawing of that parallel will teach him that we have fallen on stirring times.

That we have fallen on stirring times is the conclusion of Gervinus, not from prophecy, but from the philosophical inductions of history. The "Introduction to the History of the Nineteenth Century" has caused unusual excitement in Germany, partly because the author has a high reputation as a philosophical and far-seeing historian amongst his countrymen, partly because his philosophical "Exposition of the Course and Tendency of History" has subjected him to criminal prosecution by the government, of which his views were thought to be subversive. The work referred to is an illustration of the inductive method of foretelling the future of nations, just as Dr. Laycock's "Contributions to Proleptics" illustrate the inductive method of prognosticating the future of individuals and successive generations of men. The one weighs the developing force of ideas and the course of psychological development; the other, the force of periodic external agencies and the course of corporeal development. Both are direct and striking contrasts with the methods of divination by means of the SUPERNATURAL, which we have cursorily noticed. Gervinus notes the thunder-cloud of Russian aggression in the East, as well as the author of the "Coming Struggle," but he anticipates no battle of Armageddon, or the appearance in the field of supernatural combatants. He observes, as to Russian power:—

"Thus, as if the progress of freedom were destined to be guarded from all superficial haste, we are to all appearance more imminently than ever threatened with an universal empire, the fetter of civilization and freedom, no longer from the Catholic-Romanic nations, which are more and more being infected by the spirit of the Teutonic race, but from the Greek-Slavonic element, which, as to refinement and religion, is hostilely disposed against the centre and west of Europe. This Greek-Slavonic element consists of rude masses, dwelling in inhospitable regions, which rather suggest leaving than settling; of masses manageable in the hands of a despot and conqueror, and united by one creed, whose sole head, the Czar, is in possession of all the secular and spiritual powers; that formidable union which has been so eagerly coveted by those monarchs who aimed at establishing an universal empire in the west of Europe. What causes the danger to be still more alarming is, that the feelings of antagonism against Europe —of being one great community—of having an historical mission to regenerate over-refined society—are being fostered in the Slavonic races by a Pan-Slavonic literature and policy, which circumstances threaten to give to the collision of two opposite political principles, the character, at the same time, of a great struggle of races."

Powerful as are the autocratic and repressive forces in Europe, the democratic and expansive are greater. What Napoleon could not effect with civilized France at his back, will not be effected by Nicholas with uncivilized Russia at his. The former are dynasties only, and are already reduced to the defensive; but in history the only effective agents are those which act on the offensive. Opposed to them offensively "stand the nations in their tenacious and never-ceasing life, and the progressive spirit of history, which, without alliances, unites the nations in acting towards one end, and whose instruments are the powerful impulses of enormous masses who have no need for haste, to whom the moment has often proved fatal, but time will ever be a faithful ally."

To us, as psychologists, the future is not so hopeful. The reiterated attempts at divination by means of the supernatural indicates a stage of social development not favourable to sound religion and morals. Thus far we certainly agree with Mr. Close, who remarks—"that wonders were more frequently wrought when the revelation of God was called in question, and the worship and truth of God had been suffered to decay." In every age of the historical era—alike in the Egyptian, Assyrian, Grecian, and Roman—we find that "wonders" were the means by which the priesthood influenced the people. Pious frauds and erroneous views of the deity were always associated, and when the culminating stage arrived, marked by the utter decay of the worship and truth of God, society fell by its inherent weakness. Fraud and falsehood in matters of religion are the dry-rot of society; they canker and destroy every virtue; they extinguish all vitality. It is this which is the cardinal danger of the age, for, as Gervinus truly remarks, "universal empires do not prosper but on the ruins of decayed states, and after a complete exhaustion of the vitality of nations." The dominion of pious frauds is being established on the continent of Europe; that dominion can only rest on an *unreasoning* submission of the intellect to the priesthood; an unreasoning submission can only spring out of ignorance and superstition, and maintained by bending or breaking down all those national and social energies which the spirit of self-reliance and independence of thought fosters. A people so beat down and broken is a ready prey for the spoiler.

We will only say of the Godfreys, Beechers, &c., who would inexplicably commingle the evidences of Christianity with their visionary notions, that if Christianity have no better support than men and facts like these, it must undergo the fate of the other religious systems that have preceded it, to the end that truth may arise once more in its purity. Christian *philosophy* may, however, redeem the age from destruction, and we think that already the signs of a brighter day are dawning. In particular, a more general knowledge of physiology, and the philosophical mode of treating history on the one hand, and the increasing cultivation of meteorology on the other, give promise that a natural method of divination is arising, side by side with the supernatural. Which shall stamp its character on the age? Assyrian and Egyptian theology and philosophy were supported by fraud and superstition; they ended in the grossest ignorance and idolatry, and in the destruction of the national existence; and, as we have remarked, never was the world more given up to a multifarious system of religious fraud and falsehood than at the Christian era; the pretended supernatural was the sole basis of the established religion; it perished, consequently, by its inherent falsehood. Cato declared that he wondered how two members of the college of augurs could look each other in the face without laughing; and Cicero writ the unanswerable quiz of the whole system to which we have referred. It lived on, it is true, in a more modern dress, cherished by the ignorance and superstition of the middle ages; but this is noticeable, that nothing good, beneficial, or great—nothing, indeed, of any practical use whatever, has arisen from it.

Shall we then, in this, the middle of the 19th century of the Christian era, hamper and corrupt religion with this useless and most unprofitable system of divination and imposture—again establish "pious" frauds—again practise necromancy in the form of spirit-rappings —again profess to call demons and spirits from the vasty deep, to knock a table about—again link philosophy to the grossest fables and to wilful deceptions—again look for a thief or a lover, or a departed friend, in a magic crystal—again ask from a modern Pythian priestess, in the form of a clairvoyante girl, as to the fate of our lost heroes, or seek, by the aid of a modern sybil, to reveal the secrets of the charnel-house, and penetrate into the past not less than the future? Surely, when we consider how much the natural systems have left us amidst the wreck of ages, and how much may be attained by a diligent use and study of those systems, the age will not be tempted to abandon the safe and sure, although laborious, ways of experience and induction, for the more seductive paths of unbridled imagination and unhallowed moral influence.

Our readers will easily gather, from the foregoing remarks, that there is real danger to Christianity in the cultivation of the ideas which we have reviewed. If this be granted, then it is incumbent on the educated and more sober-minded of the clergy and laity of Christendom—not of one sect or section, but of the entire body—to stand forward and vigorously clear away from Christian doctrines and worship these Pagan doctrines of apparitions and demons.

